# Biomechanical analysis of iliac crest loading following cortico-cancellous bone harvesting

**DOI:** 10.1186/s13018-018-0822-1

**Published:** 2018-05-09

**Authors:** Paul Schmitz, Christoph Cornelius Neumann, Carsten Neumann, Michael Nerlich, Sebastian Dendorfer

**Affiliations:** 1 Clinic of Trauma Surgery, University Medical Center Regensburg, Franz-Josef-Strauss-Allee 11, D-93053 Regensburg, Germany; 2 Regensburg Center of Biomedical Engineering, Ostbayerische Technische Hochschule, Galgenbergstrasse 30, D-93053 Regensburg, Germany

**Keywords:** Bone harvesting, Autologous bone graft, Iliac crest, Fatigue fracture, Pelvis, ASIS, FEA, Biomechanical investigation

## Abstract

**Background:**

Iliac crest bone harvesting is a frequently performed surgical procedure widely used to treat bone defects. The objective of this study is to assess the biomechanical quantities related to risk for pelvic fracture after harvesting an autologous bone graft at the anterior iliac crest.

**Methods:**

Finite element models with a simulated harvest site (sized 15 × 20 mm, 15 × 35 mm, 30 × 20 mm and 30 × 35 mm) in the iliac wing are created. The relevant loading case is when the ipsilateral leg is lifted off the ground. Musculoskeletal analysis is utilized to compute the muscle and joint forces involved in this motion. These forces are used as boundary conditions for the finite element analyses. Bone tissue stress is analyzed.

**Results:**

Critical stress peaks are located between the anterior superior iliac spine (ASIS) and the anterior edge of the harvest site. Irrespective of the graft size, the iliac wing does not show any significant stress peaks with the harvest site being 20 to 25 mm posterior to the ASIS. The harvest area itself inhibits the distribution of the forces applied on the ASIS to extend to the posterior iliac wing. This leads to a lack of stress posterior to the harvest site. A balanced stress distribution with no stress peaks appears when the bone graft is taken below the iliac crest.

**Conclusion:**

A harvest site located at least 20 to 25 mm posterior to the ASIS should be preferred to minimize the risk of iliac fatigue fracture.

## Background

**Fig. 1:**
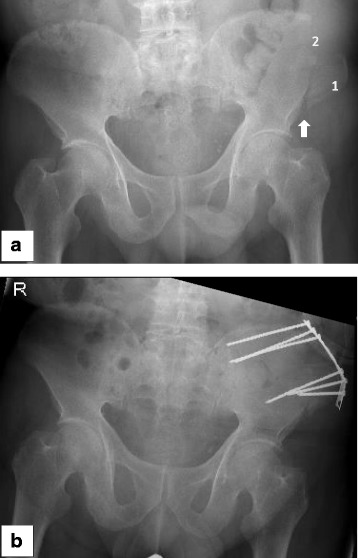
**a** Fatigue fracture of the ASIS following bone graft harvesting at the anterior iliac crest. 1 = ASIS, 2 = location of bone graft harvesting. The arrow shows the fracture side. **b** The 76-year-old patient needed a plate osteosynthesis for pain relieve and to get mobilized

Iliac crest bone harvesting is a frequently performed surgical procedure that is widely used to treat bone defects in orthopedic and trauma surgery as well as in reconstructive surgery and oral and maxillofacial surgery. Harvesting at the anterior iliac crest provides grafts with all properties required. Advantages such as fair bone quantity (cancellous bone, bicortical graft, tricortical graft, vascularized graft) when structural support is needed combined with the outstanding feature of autologous bone containing progenitor cells as well as growth factors make this relatively easy procedure to be considered as the “gold standard” [1, 2]. Nonetheless, post procedural complications such as donor site pain, gait disturbance, numbness, and fractures of the iliac crest (Fig. 1) should not be ignored [3–5]. Even though the risk for suffering an iliac crest fracture after bone harvesting seems to be rather rare (0 to 4.1%) [3, 6–9], it can alter the function for a few days up to a complete incapacity with the necessity for total bed rest or even surgical treatment [4]. Rare as well is the literature providing guidelines for the optimal location for iliac crest harvesting. Since 1995, just two experimental studies investigated this topic recommending that the graft should be harvested at least 30 mm posterior to the anterior superior iliac spine (ASIS) and the length of the graft should not exceed more than 30 mm to prevent fatigue iliac crest fracture [4, 10].

The objective of this investigation is to assess the risk for fatigue iliac crest fracture following cortico-cancellous bone harvesting at the anterior iliac wing.

## Methods

### Creating the FE models

Using the software Simpleware (ScanIP 7.0 +FE), a segmented 3D model of the ilium is created from a computer tomography (CT) provided by the University Regensburg Medical Center. Due to the nature of the study based on a CT scan that was performed for another reason than the finite element analysis (FEA) without need for any further individual data, the “Independent Ethics Committee of the Faculty of Medicine” at the University Regensburg Medical Centre confirmed that an ethics opinion in accordance with the 1964 Helsinki Declaration is not necessary (institutional review board number 18-180-0000). The joints between pelvis and sacrum are stiffened to simplify the 3D modeling and the calculation. The depicted MRI (Fig. 4) and X-rays (Fig. 1 and Fig. 6) originate from patients that were treated due to a fatigue fracture of the ASIS following bone graft harvesting at the anterior iliac crest. The images were performed for another reason than the FEA and were included retrospectively in this investigation.

FEA is performed using Ansys (Ansys Classic, Mechanical APDL, Release 15.0). To reach convergence of the FEA, the h-method was applied to the FE model and a mesh quality of − 46 (− 50: coarse, 50: fine) was set in Simpleware. The mesh algorithm was set to “+FE Free” with tetrahedral elements. In order to show stress distribution over the whole pelvis, the quality of the mesh is set to coarse.

**Fig. 2:**
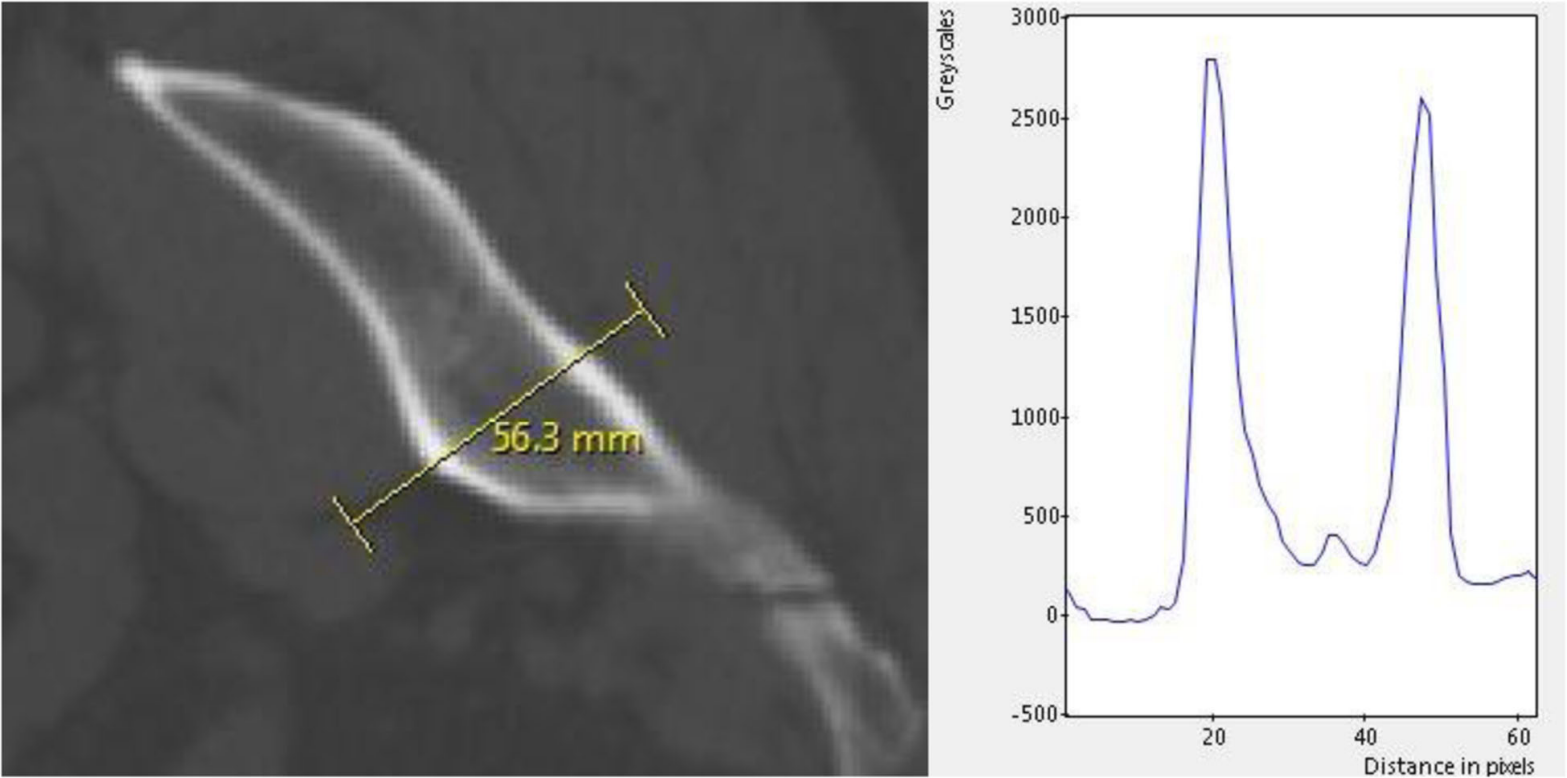
Exemplary grayscale values of the ilium from the CT scan

Material properties for cortical bone were set to fixed values, whereas cancellous bone properties have been defined according to greyscale values given in the CT scan (Fig. 2). The following equations were used for the mapping:

1. Density *= a + b ×* GS
  *a* = 4.692e+002
  *b* = 3.077e−001
  GS = grayscale value
2. Young’s modulus *= c ×* Density^*d*^
  *c* = 3.389e−011
  *d* = 6.843

The range of material properties is displayed in Table 1.

**Table 1:** Material properties of cancellous as well as cortical bone with their related greyscale values used in this study

	Grayscale [−]	Density [kg/m^3^]	Young’s modulus [MPa]	Poisson’s ratio [−]
Cancellous bone	100–500	500 … 700	100 … 1000	0.2
Cortical bone	500–3000	1500	10,000	0.3

**Fig. 3:**
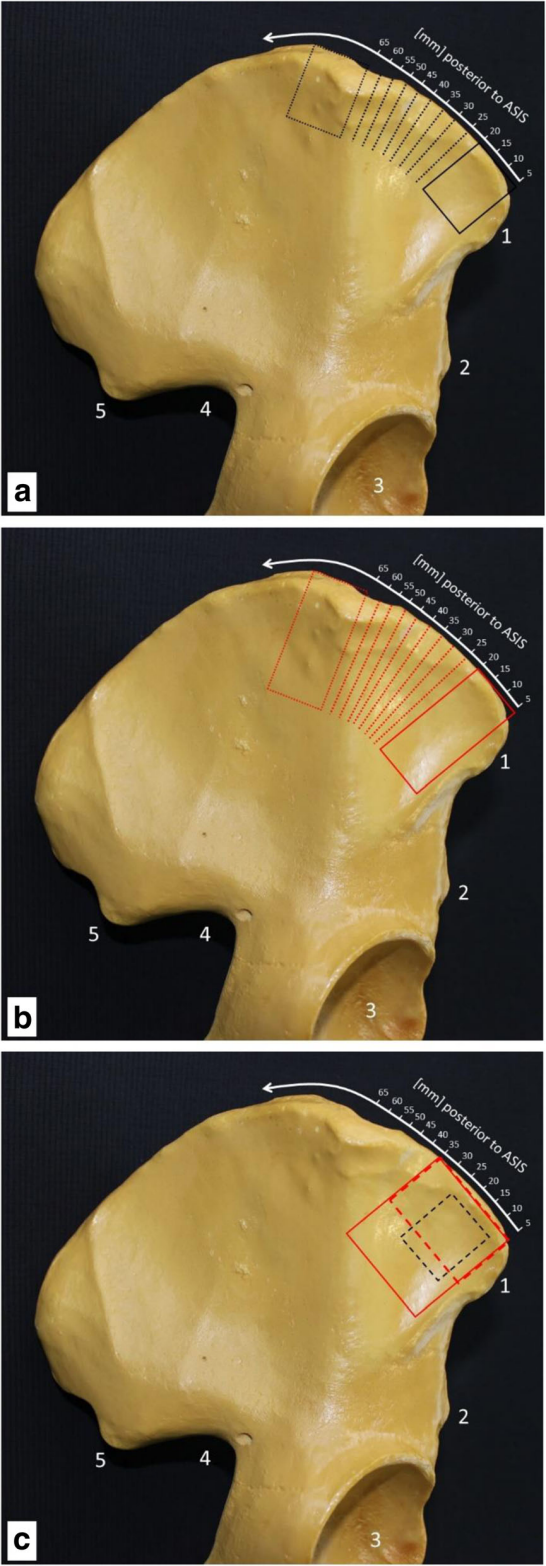
Right iliac bone: 1 = anterior superior iliac spine (ASIS), 2 = anterior inferior iliac spine, 3 = acetabulum, 4 = sciatic notch, 5 = posterior inferior iliac spine. Bone graft harvest site of 15 × 20 mm (**a**, black rectangle) and 15 × 35 mm (**b**, red rectangle). Dotted lines and dotted rectangles = the position of the harvest side was drafted beginning 5 mm posterior to the ASIS along the iliac wing. **c** Bone graft harvest site of 30 × 20 mm (red dashed rectangle) and 30 × 35 mm (red rectangle) 5 mm posterior to the ASIS. Black dashed rectangle = cavity of 15 × 20 mm was taken 5 mm below the iliac crest and 10 mm posterior to the ASIS

The sizes of cutouts simulating the harvested bone material of two studies with 10 different positions are 15 mm × 20 mm (Fig. 3a) and 15 mm × 35 mm (Fig. 3b). The first starting point of the gaps in the iliac wing is 5 mm posterior to the ASIS. Nine more cavities are located along the iliac crest by increments of 5 mm. Two additional gaps in the iliac wing 5 mm posterior to the ASIS with a size of 30 mm × 20 mm and 30 mm × 35 mm (Fig. 3c) are analyzed in this study. Furthermore, one model with a 15 mm × 20 mm cavity (Fig. 3c) was taken 5 mm below the iliac crest and 10 mm posterior to the ASIS. The models are created with a Python code in Simpleware.

### Applying the musculoskeletal forces onto the FE model and performing finite elements analysis (FEA)

**Fig. 4:**
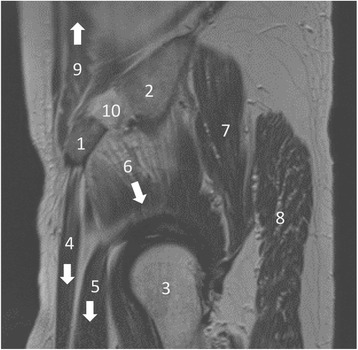
MRI of a pelvis and the hip joint in T2 sagittal reconstruction: 1 = ASIS, 2 = iliac crest, 3 = femoral head, 4 = sartorius muscle, 5 = femoral rectus muscle, 6–8 = minor, medium, and greater gluteal muscles, 9 = abdominal muscle, 10 = location of bone graft harvesting. The arrows show the traction of muscle forces that provoke a fatigue fracture of the ASIS [13]

The relevant loading case for fatigue fractures of the anterior iliac crest is when the leg on the harvested side is lifted of the ground. Then the muscles attached to the ASIS (Fig. 4) as well as the joint forces apply the highest load onto the anterior ilium during the gait process. To know which forces have to be applied onto the ilium in the FEA, a gait analysis of a healthy skeletal mature individual without iliac crest bone harvesting is performed with the musculoskeletal simulation software AnyBody (AnyBody 6.0) [11]. Gait is analyzed using a predefined musculoskeletal model (MoCap_FullBody, AnyBody Managed Model Repository). Muscle and joint reaction forces are analyzed and transferred to the finite element model. The muscle attachments are simulated with BEAM4 elements. Hip joint forces as well as the forces applied on the ASIS are manually integrated onto the model. Rigid boundary conditions are applied to Sacrum (S1).

### Outcome variables

Von-Mises stress distribution is analyzed.

## Results

**Fig. 5:**
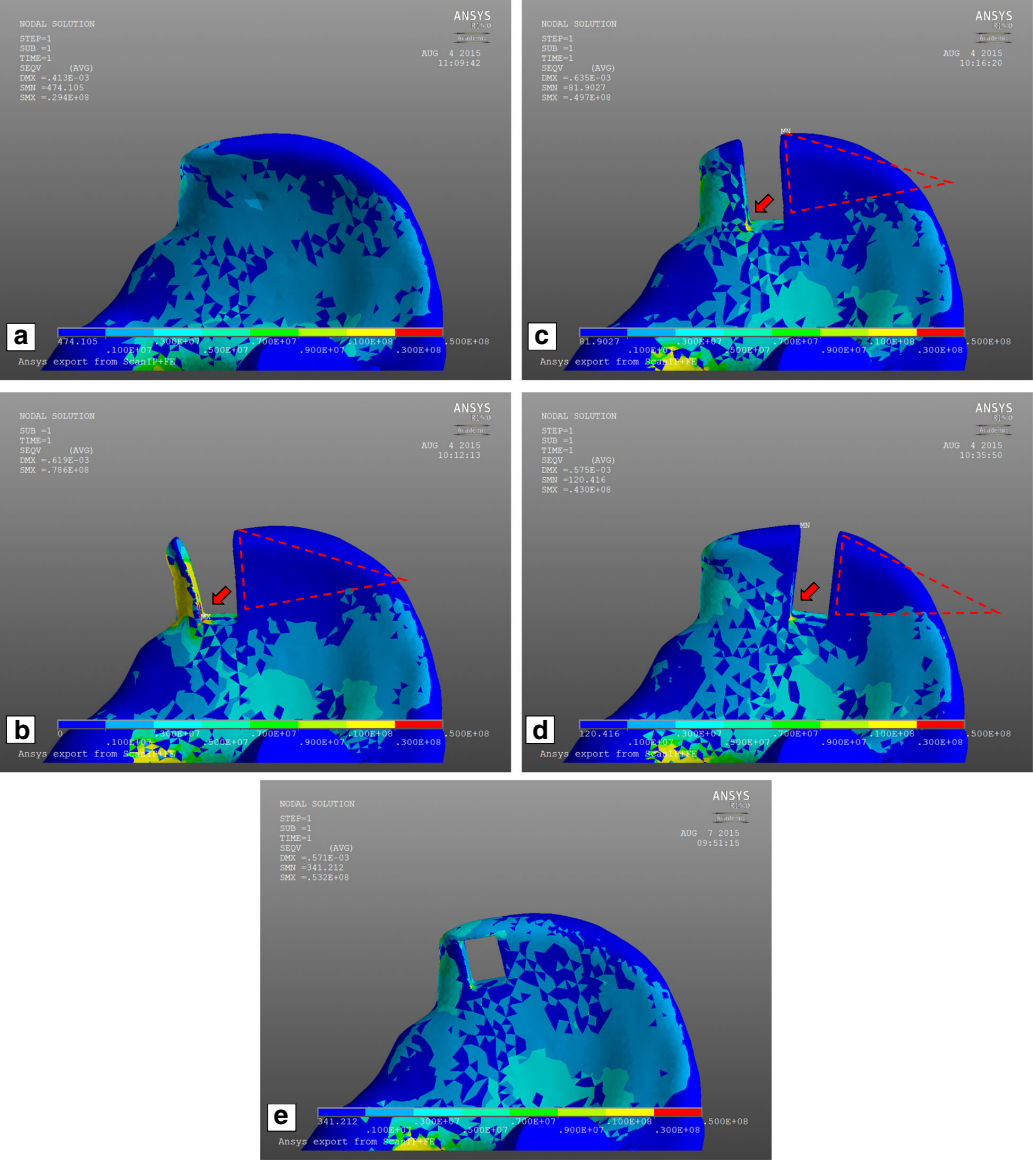
**a** Right iliac wing without bone graft harvest. **b**–**d** Right iliac wing with simulated bone graft harvest site of 15 × 35 mm with the cutout 5 mm (**b**), 15 mm (**c**), and 35 mm (**d**) posterior to the ASIS. **e** Right iliac wing with 15 × 20 mm cutout 10 mm posterior to the ASIS and 5 mm below the iliac crest

The results of the FEA are explained by showing the stress distribution on the right iliac wing of five exemplary analysis. All stress values are in Pascal. Well-balanced stress values along the iliac wing and the absence of stress peaks are present for an intact iliac wing (Fig. 5a). Multiple stress peaks between the ASIS and the cutout can be detected when the gap in the iliac crest is placed 5 mm posterior to the ASIS (Fig. 5b–d). A lack of stress occurs along the iliac wing posterior to the gap. This area is of a triangular shape with one side being the edge of the cutout and one side being the iliac crest (Fig. 5b–d).

By successively placing the gap further away from the ASIS, the stress peak between the ASIS and the anterior edge of the cutout is notably reduced and the lack of stress along the iliac wing posterior to the cutout diminishes successively as well (Fig. 5c, d).

The stress distribution along the iliac wing is equal irrespective of the cutout size (15 × 20 mm versus 15 × 35 mm) but with a deeper cavity; stress values between the ASIS and the gap are notably higher. Contrary to the 15 × 20 mm cutout, a stress peak remains between the ASIS and the anterior edge of the 15 × 35 mm cutout when this is placed even 35 mm posterior to the ASIS. Furthermore, the area of the lack of stress posterior to the gap is significantly bigger for the 15 × 35 mm gap than for the 15 × 20 mm gap.

With a cutout that leaves the iliac crest intact, the stress distribution on the iliac wing between the cavity and the ASIS is notably relieved (Fig. 5e). There are no stress peaks on the iliac wing. The stress distribution posterior to the cutout is balanced contrary to every other model with a gap in the iliac crest.

## Discussion

Fractures of the iliac wing following autologous bone graft harvesting seem to be with less than 5% a rare complication [3, 6–9] in comparison to the estimated overall morbidity rate of 19.37% [5]. In 2/3 of the cases, these fractures are fatigue fractures of the ASIS after harvesting the anterior iliac crest [12]. One of the main reasons for postoperative fractures of the ilium seems to be the sudden contraction of the muscles attached to the ASIS, sartorius muscle, and tensor fascia muscle [13]. To reestablish the function of these muscles as well as to induce pain relief to the patient in 16.6%, a surgical stabilization (Fig. 1) has to be performed [12].

**Fig. 6:**
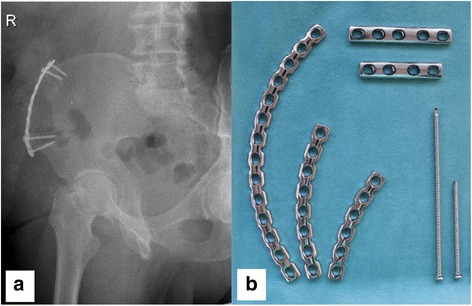
**a** X-ray (right pelvis ala-view) of a 73-year-old patient 8 years after bone harvesting (vascularized iliac crest) and simultaneous plate osteosynthesis to prevent a fatigue fracture. **b** Anatomically shaped low profile small fragment plates and cortical screws (optionally angular stable) that can be used to reconstruct the iliac crest following bone graft harvesting

Different surgical techniques including autologous rib transplantation [14] as well as xenogenous cancellous allografts [15] are proposed to restore the bone defect after iliac crest harvesting in order to prevent local complications. In our opinion, the method to cover the iliac gap by fixation of a plate [16, 17] (Fig. 6) is an adequate way to prevent fatigue fracture of the ASIS even though there is no clinical or biomechanical study to which factor the risk of an iliac crest fracture is reduced by it.

Looking at the result of our biomechanical FEA, the stress distribution along the iliac wing varies from critical to stress-relieved depending on location and size of the harvested bone. As expected, stress peaks occur especially between the ASIS and the anterior edge of the harvest site. The closer the harvest site is placed to the ASIS and the deeper the size of the harvest site is performed, the higher the stress peaks arise. The danger of a small crack spreading out from the anterior edge of the harvest site to the front of the ilium and thus causing a fracture of the anterior iliac crest becomes obvious.

Irrespective of the harvest size (15 × 20 mm versus 15 × 35 mm), the iliac wing does not show any significant stress peaks when the harvest site starts 20 to 25 mm posterior to the ASIS. Proving Hu et al. [4] right that bone graft taken 30 mm posterior to the ASIS significantly reduces the risk of anterior iliac crest fractures, this study even suggests going as close as 20 to 25 mm to the ASIS without a mentionable increase of the risk for anterior iliac crest fractures. Lengthening the harvest area from 15 to 30 mm does not notably impact the stress distribution between the ASIS and the cutout (data not shown). Nonetheless, the harvest site should be chosen as small as possible since many abdominal muscles are attached to the iliac crest and a herniation of visceral organs can be prevented.

Creating a gap in the iliac crest by bone harvesting interrupts the force flow that usually spreads out from the ASIS along the iliac crest to the posterior iliac wing. This way, a triangular shaped area of low stress posterior to the harvested site emerges (Fig. 5). Referring to Wolff’s law without the application of stress, this part of the iliac wing will become weaker [18], thus increasing the risk for a consecutive fracture at this area years after bone graft harvesting. Furthermore, it might be a reason for the most frequent complication caused by bone harvesting at the iliac crest: the persisting local pain [3–6]. Whereas recent investigations show that iliac crest harvesting does not result in an increase of acute pain or narcotics consumption [19, 20], Sasso et al. point out that donor site pain remains a significant postoperative problem even years after treatment [21]. To avoid adverse events caused by autologous iliac crest, harvesting allografts as well as synthetic bone grafts always has to be considered as an alternative even though an evidence for superiority is not given yet [22, 23].

To eliminate the stress peaks between the ASIS and the harvest site as well as to preserve a force transmission from the ASIS to the posterior iliac wing, the bone graft could be harvested below the iliac crest (Fig. 5e). Having a continuous and uninterrupted iliac crest balances the stress distribution on the iliac wing with the implication that just a bicortical graft can be extracted. If a tricortical bone graft is needed, rebuilding the iliac crest with the fixation of a plate over the harvest area (Fig. 6) might recreate a balanced force distribution along the iliac wing. It should relieve the stress applied on the anterior iliac wing between the ASIS and the harvest site as well as eliminate the lack of stress in the area posterior to the harvest site.

*Discussion of the model:* Using 3D modeling and finite element analysis provides the opportunity to investigate various ways of iliac crest harvesting as well as different sizes of bone grafts in one model and with low costs. However, several limitations are inherent using these kinds of simulations. First of all, the gait analysis is simplified since it is of a healthy skeletal mature individual without iliac crest bone harvesting. A gait analysis of a harvested patient certainly would be different either due to pain or due to biomechanical reasons. Respectively, the distribution of force over the ilium would be different too.

Analyzing the influences on the iliac wing when maximum stress is applied by the muscles attached to the pelvis and by the forces that occur at the hip joint during gait analysis leaves possible outside influences out of consideration. Load applied on the pelvis by torsional mass is not taken into account. The points of attachment of muscles might vary from their real location since the forces are exported from an ideal model in AnyBody. Since this study focuses on the relative stress distribution on the iliac wing rather than the absolute stress values, the bone age of the used CT scan is not of significant relevance.

## Conclusion

In conclusion, there are three options to significantly decrease the risk of fatigue iliac crest fracture following bone graft harvesting from the anterior iliac wing. First of all, leaving the iliac crest intact by harvesting below it significantly relieves stress peaks between the ASIS and the harvest site. It balances the stress distribution along the iliac wing as well. Secondly, a rather longer than deeper bone graft should be preferred from the biomechanical point of view. However, respecting the anatomy with many abdominal muscles attached to the iliac crest, a proper balance between length and depth of the bone graft needs to be found. Most of all a harvest site located at least 20 to 25 mm posterior to the ASIS should be preferred to minimize the risk of iliac fatigue fracture. Even though this study has not proven that a reconstruction of the iliac crest with plate fixation over the harvest area will prevent a fatigue iliac wing fracture, the authors are convinced of it.

## Abbreviations

ASIS: Anterior superior iliac spine
CT: Computer tomography
FEA: Finite element analysis
MRI: Magnetic resonance imaging
